# Publisher Correction: Plate-nanolattices at the theoretical limit of stiffness and strength

**DOI:** 10.1038/s41467-020-16326-1

**Published:** 2020-05-08

**Authors:** Cameron Crook, Jens Bauer, Anna Guell Izard, Cristine Santos de Oliveira, Juliana Martins de Souza e Silva, Jonathan B. Berger, Lorenzo Valdevit

**Affiliations:** 1 Department of Materials Science and Engineering, University of California, Irvine, CA USA; 2 Department of Mechanical and Aerospace Engineering, University of California, Irvine, CA USA; 3 Institute of Physics, Martin-Luther-Universität Halle-Wittenberg, Halle, Germany; 4 Nama Development, LLC, Goleta, CA USA; 5 Materials Department, University of California, Santa Barbara, CA USA

**Keywords:** Metamaterials

Correction to: *Nature Communications* 10.1038/s41467-020-15434-2, published online 27 March 2020.

The original version of this article contained an error in Fig. 3d, in which the plot border and the x- and y-tick marks of Fig. 3d were missing.

This error has now been corrected in both the PDF and the HTML versions of the Article.

