# Supplementary material for: Plate-nanolattices at the theoretical limit of stiffness and strength
Source: Nat Commun. 2020 Mar 27;11:1579. doi: 10.1038/s41467-020-15434-2 (PMC7101344; doi:10.1038/s41467-020-15434-2)
Supplement: Supplementary file 1 — Supplementary Information [file 41467_2020_15434_MOESM1_ESM.pdf]

Supplementary Information:

Plate-Nanolattices at the Theoretical Limit of Stiffness and Strength

Cameron Crook, et al.

Supplementary Note 1: Pyrolytic carbon cubic+octet plate-nanolattice manufacturing strategy

Cubic+octet plate-nanolattices were fabricated by a two-photon-polymerization direct laser writing (TPP-DLW) and pyrolysis[1] fabrication route (Supplementary Figure 1). Samples were printed atop a silicon wafer with a drop of IP-Dip resin into which the writing objective was inserted from below, also termed Dip-in Laser Lithography (DiLL). After printing, samples were developed in a PGMEA bath followed by an IPA bath and then dried in a critical point dryer (CPD). Once developed, samples were pyrolyzed to convert the polymer to pyrolytic carbon.

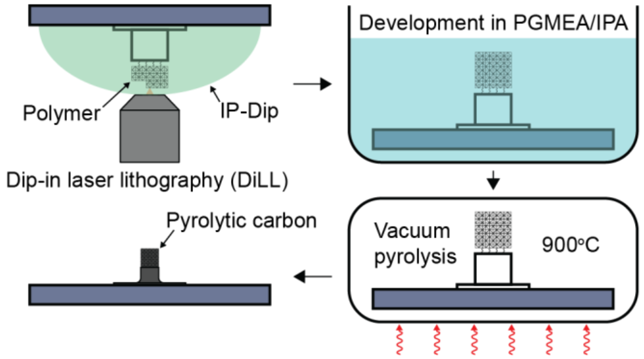

Supplementary Figure 1 | Illustration of two-photon-polymerization-direct laser writing (TPP-DLW) and pyrolysis process for manufacturing of pyrolytic ceramic nanolattices.

TPP-DLW and pyrolysis has been successfully employed for open-cell nano-architectures, like beam-lattices[1–3], whereby entire structures can be printed in a simple 3D trajectory pattern with a fixed set of process parameters. In contrast, the complexity of plate-nanolattices imposes several manufacturing challenges, including precise wall thickness and constituent material property control, retention of high surface quality while printing at the TPP-DLW resolution limit in a layer-by-layer pattern, and accommodation of pyrolysis-induced shrinkage with ~3 times larger structures compared to same-feature-size beam-lattices of a given relative density. To resolve the above challenges, we have developed a plate orientation specific TPP-DLW strategy, encompassing a writing parameter optimization procedure using SEM-based wall thickness and surface quality calibration, micro-Raman spectroscopy-measured microstructural characterization and optimization of support pillars and springs.

Supplementary Figure 2 illustrates the hatching strategies adopted to print horizontal and vertical cubic walls of the same thickness with the correct thickness ratio to the octet walls, while achieving the smallest possible feature size and hence structure sizes. This ensures manufacturability within the limited print volume and minimal pyrolysis-induced shrinkage deformations in addition to the best possible exploitation of material-strengthening size-effects. The wall-specific hatching strategy, necessitated by the ellipsoidal voxel shape inherent to TPP-DLW, required thickness control of single voxel-thick horizontal cubic and octet walls by laser average power, while the vertical cubic wall thickness was controlled by the hatching distance and number of hatched lines.

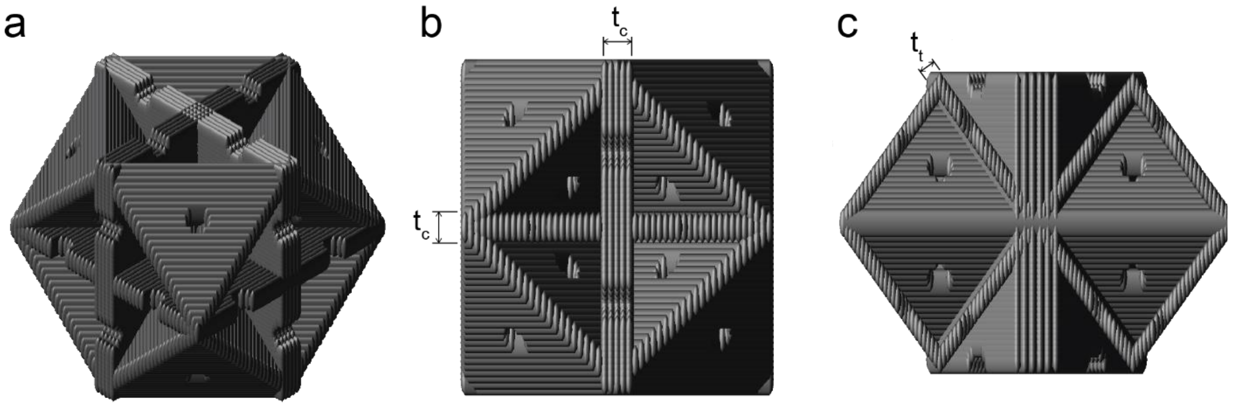

Supplementary Figure 2 | Wall orientation specific hatching strategies print cubic+octet plate-lattices with maximal surface quality and smallest possible feature sizes. Renderings of cubic+octet plate-lattice TPP-DLW print strategies of a unit cell (a-c) showing that octet and horizontal cubic walls are composed of single voxel-thick lines and vertical cubic walls are composed of many closely hatched lines. Wall thicknesses are denoted as t_t and t_c for octet and cubic walls, respectively. Note that slicing, hatching distances, and number of vertical hatched lines are coarsened to easily view the print strategy.

The degree of conversion and hence the mechanical properties of TPP-DLW-derived polymer and pyrolytic carbon largely vary depending on the TPP-DLW writing parameters,[4] such as writing speed (*v*), laser average power (P) as well as hatching (hd) and slicing distance (sd). For the best compromise between structure quality and print time, a *v* of 5,000 µm/s and *sd* of 0.05 µm were chosen. Before selecting specific P and hd based on the desired wall thicknesses, the degree of conversion (DC) of individual polymer unit cell plate orientations following a post-print bake were measured by micro-Raman spectroscopy for a range of writing parameters within the printable range. The results of these measurements (Supplementary Figure 3a) revealed that the DC largely clustered around 60%. Wall thickness data of fixed hatching distances for the cubic walls (Supplementary Figure 3b) was then measured from polymer calibration structures (Supplementary Figure 3c) also used to assess the relative surface quality and dimensional reproducibility.

Writing parameters, laser average power, hatching distance and number of hatched lines were then selected within the parametric sweep range of the Raman and wall thickness calibration data in order to provide the best surface quality, smallest feature dimensions, approximately identical DC and correct wall thickness ratio (Supplementary Figure 3b). The final hatching distance of the vertical walls was doubled from 0.05 µm to 0.1 µm and the number of hatched lines was halved to 6 to reduce the printing time without noticeably changing the DC or vertical wall dimensions. This process resulted in the final writing parameters: 16 mW *P* for octet walls, 12 mW *P*, 6 hatched lines and 0.1 µm *hd* for vertical cubic walls, and 15.25 mW *P* and 0.05 µm *hd* for horizontal cubic walls. The resulting polymer wall thicknesses of the octet and cubic walls were 0.59 µm and 1.01 µm, respectively. The Raman spectra of Supplementary Figure 3d and Table 1 show excellent overlap, indicating that identical microstructures were obtained for all wall directions using the final writing parameters.

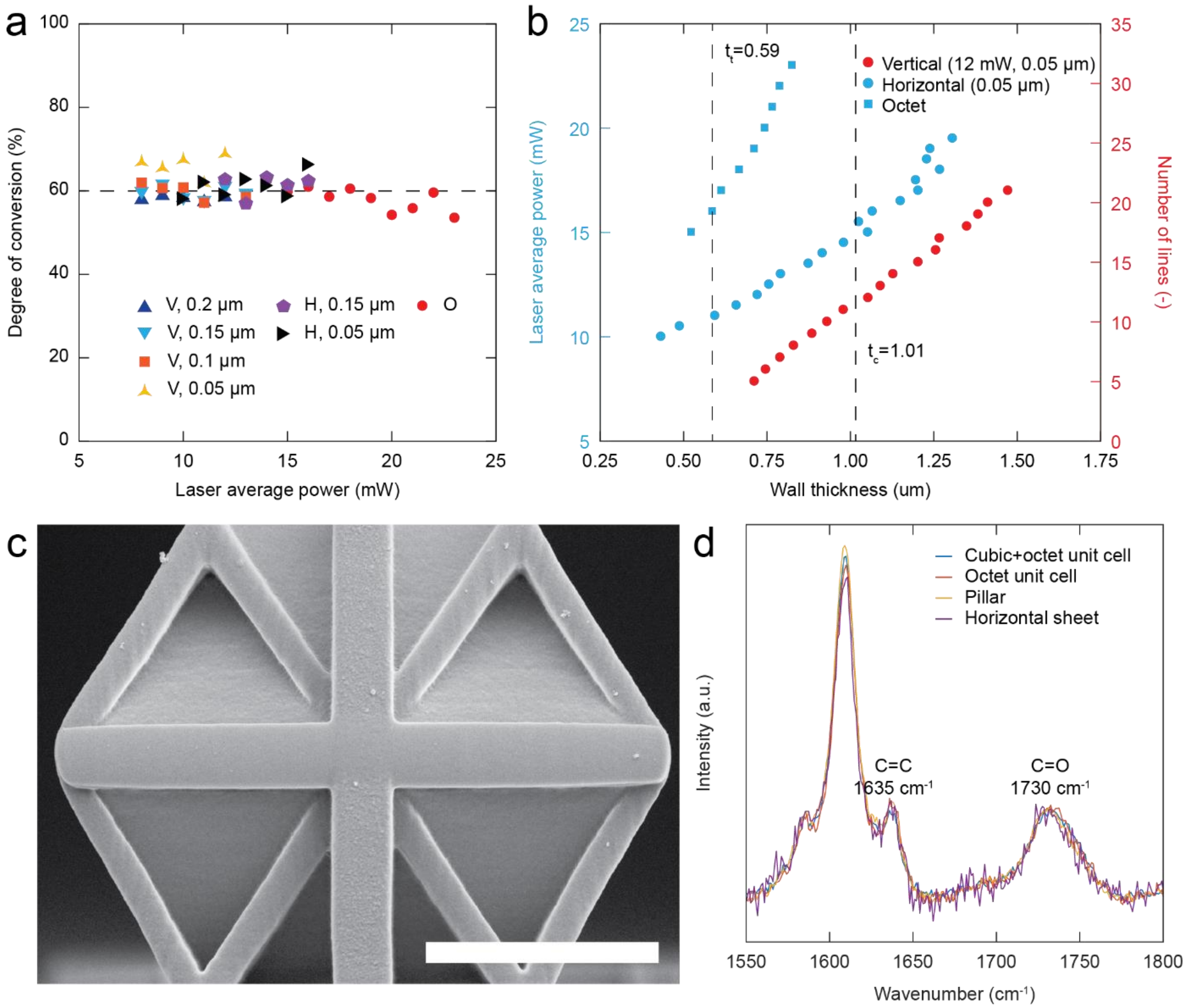

Supplementary Figure 3 | Writing parameter optimization encompassing degree of conversion and wall thicknesses measurements of individual plate orientations to produce high surface quality plate-nanolattices with isotropic constituent material and correct cubic-to-octet wall thickness ratio. Micro-Raman measurements of polymeric of horizontal cubic (H), vertical cubic (V), and octet (O) plate orientations (a) show clustering of degree of conversion near 60%. Wall thickness calibration data controlled by laser average power (blue) or number of hatched lines (red) (b). Dashed lines indicate the chosen octet (t_t) and cubic (t_c) wall thicknesses. SEM micrograph of a wall thickness calibration structure (c) which was printed with a cut-open along the (110) plane shows walls have smooth surface finish. Scale bar is 5 µm. Raman spectra of polymeric cubic+octet and octet plate-lattice unit cells, pillar printed with the vertical wall parameters and single voxel-thick sheet printed with the horizontal wall parameters (d) show identical degree of conversion. Source data are provided as a Source Data file.

Lastly, the polymeric structures had to accommodate the extreme shrinkage of pyrolysis with minimal surface deformations. As introduced in previous work[1], pre-pyrolysis TPP-DLW-printed polymer cubic+octet plate-microlattices were supported atop springs attached to pillars to decouple them from the substrate (Supplementary Figure 4). However, shrinkage during pyrolysis of polymers notably increases with increasing surface area and decreasing size. Compared to previously reported beam-lattices, the considerably larger plate-lattice dimensions exacerbated the shrinkage mismatch between support pillar and lattice. We developed a coarse pillar hatching strategy which introduced porosity into

the support pillars reducing this mismatch to about 7%. Uniform shrinkage of the porous round pillars was ensured by rotating adjacent sliced layers by 30° from one another in a clockwise fashion as TPP-DLW lines tend to shrink along the writing direction. The aspect ratio of the support pillar, 0.85, was optimized to ensure a flat post-pyrolysis pillar top surface. Support pillar optimization in conjunction with carefully selected spring locations and dimensions reduced deformation during pyrolysis to a minimum.

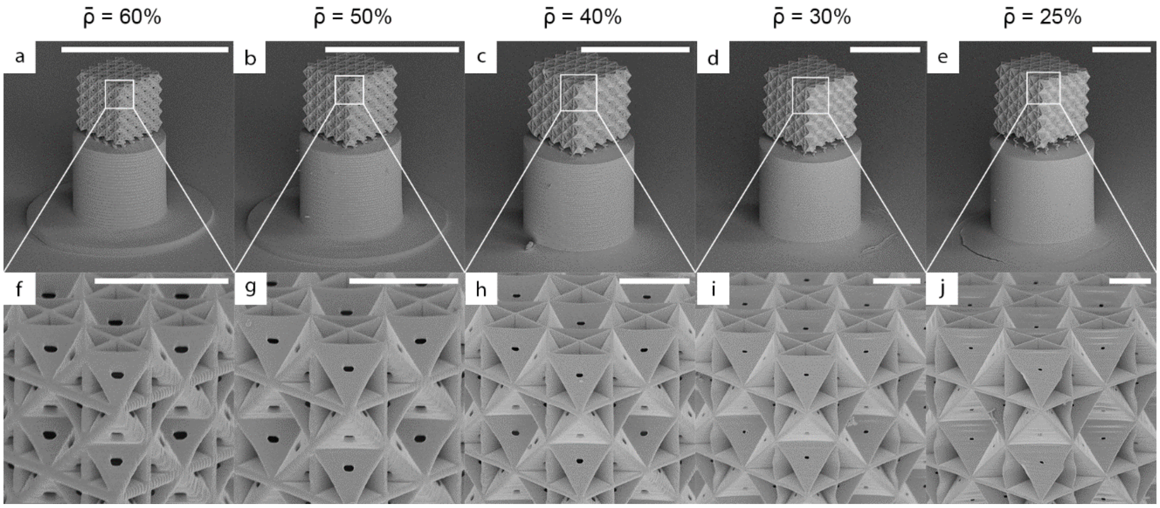

Supplementary Figure 4 | SEM micrographs of pre-pyrolysis polymer plate-nanolattices show near perfect lattices. Pre-pyrolysis polymer lattices are suspended atop pillars by coiled springs (a-b). Close ups of the unit cells (f-k) show that walls begin to warp slightly below $\bar{\rho} = 30\%$, but are otherwise perfect. Scale bars are 100 µm (a-e) and 5 µm (f-k).

Raman spectra were also collected from pyrolytic carbon pillars, printed with the writing parameters of the vertical cubic walls, and plate-nanolattices. The degree of graphitization of the pillars and plate-nanolattices appear identical in Supplementary Figure 5 and Table 2. Therefore, the constituent material of both have identical microstructures and thus mechanical properties.

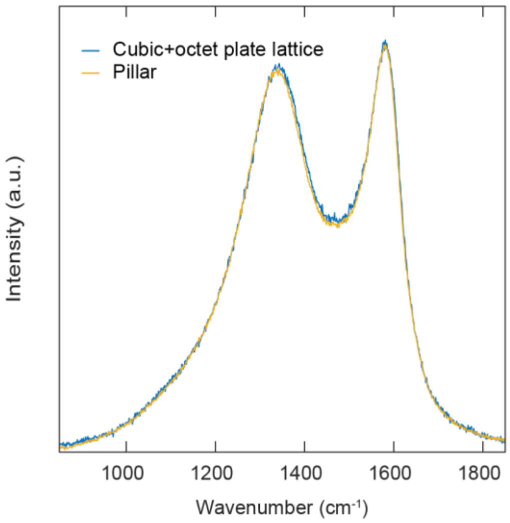

Supplementary Figure 5 | Raman spectra of pyrolytic carbon cubic+octet plate-nanolattices and micro-pillars indicate identical degree of graphitization and hence identical microstructures. Source data are provided as a Source Data file.

Supplementary Table 1 | Raman spectroscopy results of polymer structures

	C=C (1635 cm^{-1})		C=O (1730 cm^{-1})		
Structure	Peak Intensity (a.u.)	Fit Error (%)	Peak Intensity (a.u.)	Fit Error (%)	Degree of Conversion (%)
Cubic+octet plate-nanolattice unit cell	20500	3.68	47750	5.47	59.95
Octet plate-nanolattice unit cell	79740	2.16	191300	4.69	61.04
Square pillar (vertical cubic wall)	311400	2.31	680300	3.94	57.22
Single voxel thick sheet (horizontal cubic wall)	34480	3.31	78230	9.11	58.81

Supplementary Table 2 | Raman spectroscopy results of pyrolytic carbon structures

	R2		Half-width-half-max (HWHM) of D1	
Structure	Average (-)	Rel. Standard Deviation (%)	Average (-)	Rel. Standard Deviation (%)
Cubic+octet plate-nanolattices	0.80	5.7	104.57	9.2
Micro-pillars	0.83	3.6	110.42	6.7

Supplementary Table 3 | Measurement parameters used in the X-ray microscopy CT experiments

Sample Relative Density	Projection exposure time (s)	Field of view *	Voxel size
**60%**	100	HRES (16 μm^3)	16 nm
**40%**	60	LFOV (64 μm^3)	64 nm
**30%**	60	LFOV (64 μm^3)	64 nm

HRES: High resolution mode
LFOV: Large field of view mode

Supplementary Note 2: Experimental characterization of pyrolytic carbon plate-nanolattices

To determine the constituent material properties of our plate-nanolattices a total of 8 circular pyrolytic carbon micro-pillars were *in situ* mechanically tested in uniaxial compression under identical conditions as the cubic+octet plate-nanolattices, i.e. using a loading-unloading cycle at 7% strain (Supplementary Figure 6). Considering the process parameter dependency[4] of TPP-DLW-derived structures, the same printing strategy of the vertical cubic walls of the plate-nanolattices was used to print the micro-pillars to most closely match the actual constituent properties. To reliably correct for substrate and system compliance via digital image correlation (DIC) and to reduce surface roughness due to the unidirectional hatching of the circular pillars, diameters of 6 µm were chosen. An aspect ratio of 3 between the height and diameter[5] was used. Stiffnesses and yield strengths between 37 and 44 GPa and 2.2 and 2.7 GPa, respectively, were determined from DIC corrected stress-strain curves. Post-yield buckling prevented maximum compressive strength measurement, however maximum stresses were on the order of reported compressive strengths[6] of TPP-DLW-derived pyrolytic carbon.

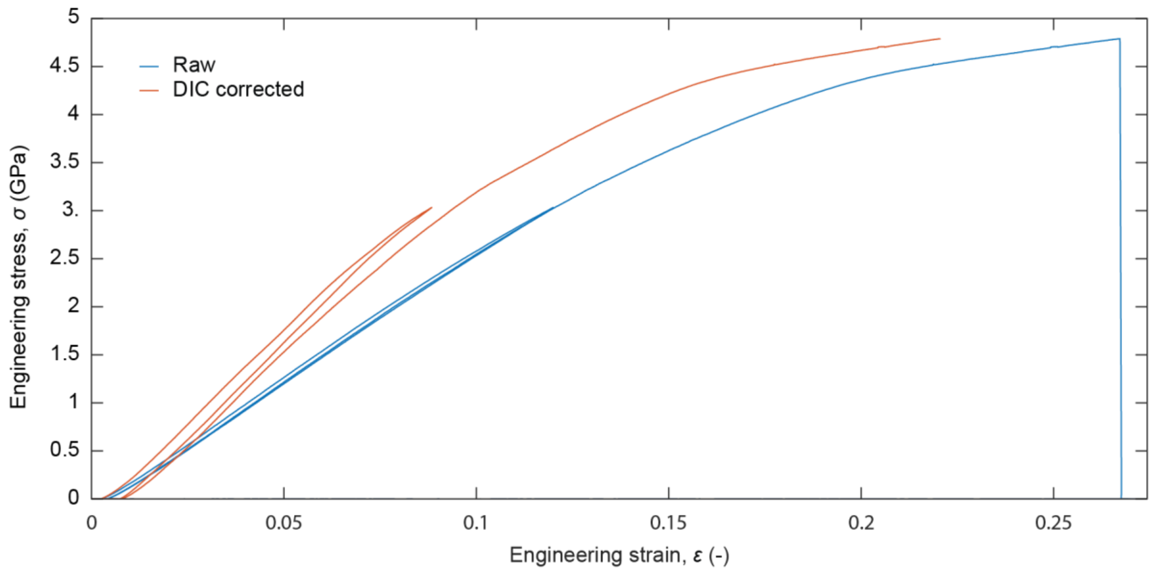

Supplementary Figure 6 | Compression experiment of pyrolytic carbon micro-pillar. As measured, raw, and digital image correlation (DIC) corrected compressive stress strain curves. Source data are provided as a Source Data file.

Supplementary Figure 7 shows the strain at failure of our plate-nanolattices, which undergo elastic-plastic deformation ($\bar{\rho} \geq 37.5\%$), decomposed into their elastic and plastic components. The highest relative density plate-nanolattice ($\bar{\rho} = 57.5\%$) had a failure strain of approximately 21.9%, comparable to pyrolytic carbon nanoarchitectures of the same relative density[1,3,7]. As reported for many brittle beam-lattices[2,3,8], we found the failure strain of plate-nanolattices with relative densities above 37.5% to decrease with decreasing $\bar{\rho}$. Given that stiffness and yield strength both scaled nearly linearly with density, the yield strain was independent of $\bar{\rho}$. Deformation until failure was predominantly elastic. Finite element analysis (Figure 5) indicated that the failure strain-relative density dependency is related to post-yield buckling. Given that pyrolytic carbon at this scale only shows a modest change in stiffness between elastic and plastic deformation, the elastic buckling simulations well approximate the plastic buckling strength. In good agreement with the measured compressive strength, the computed buckling

strength exceeds the yield strength above $\bar{\rho} = 37.5\%$ and rapidly increases with $\bar{\rho}$. Simultaneously, the failure strain increases, causing the found dependency with $\bar{\rho}$. At low relative densities ($\bar{\rho} < 37.5\%$), where structures fail in a layer-by-layer fashion, meaningful failure strains may not be defined, because structures can be progressively deformed until densification. Compression experiments were terminated after failure of the first unit cell layer in our experiments.

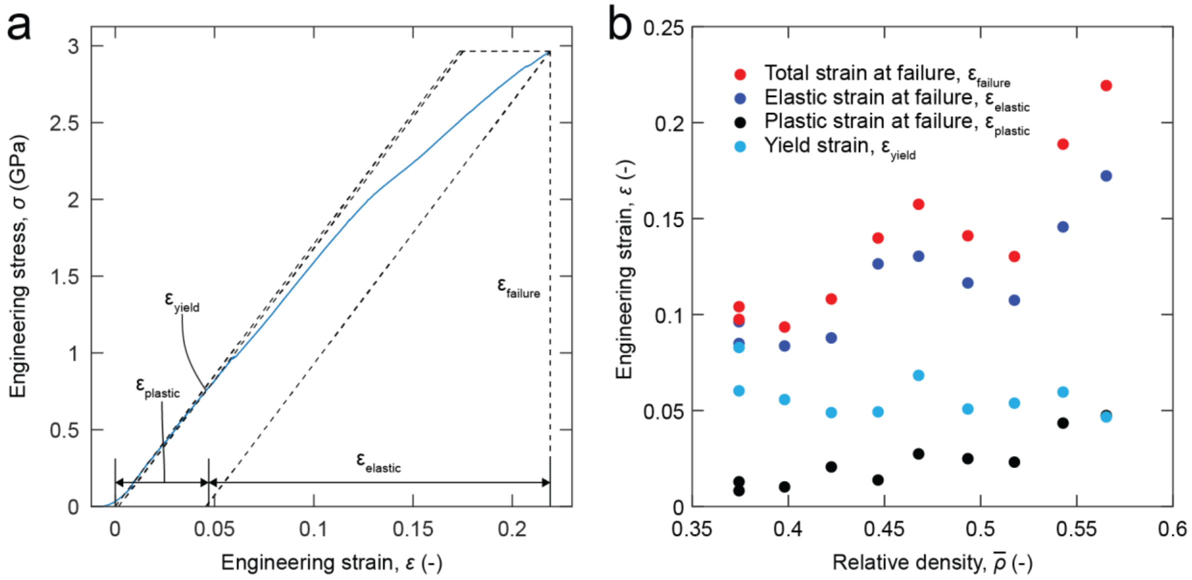

Supplementary Figure 7 | Failure strains of high-$\bar{\rho}$ pyrolytic carbon cubic+octet plate-nanolattices are predominantly elastic and decrease with decreasing relative density. Digital image corrected stress-strain response of the $\bar{\rho} = 57.5\%$ specimen with strain components (a). Strain components at failure depending on the relative density (b). Source data are provided as a Source Data file.

Supplementary Figure 8 shows Ashby charts of stiffness and strength, which are normalized by the constituent material properties. Unfortunately, it is often challenging to extract reliable constituent material properties of architected materials from literature, particularly in the case of micro- and nano-architected structures exploiting size effect strengthening, which represent the vast majority of architected materials. Hence, in order to provide meaningful comparisons, we only report data for TPP-DLW-derived pyrolytic carbon architectures. While limited to a single material system, the topologies we present nevertheless include the most relevant beam-architectures, like octet and isotropic trusses, as well as shell spinodal topologies. The cubic+octet topology of our plate-nanolattices can clearly be seen to outperform all other available architectures in both strength and stiffness. Absolute strength, stiffness and density values were normalized by 2.5 GPa, 62 GPa and 1.4 g/cm³, respectively.

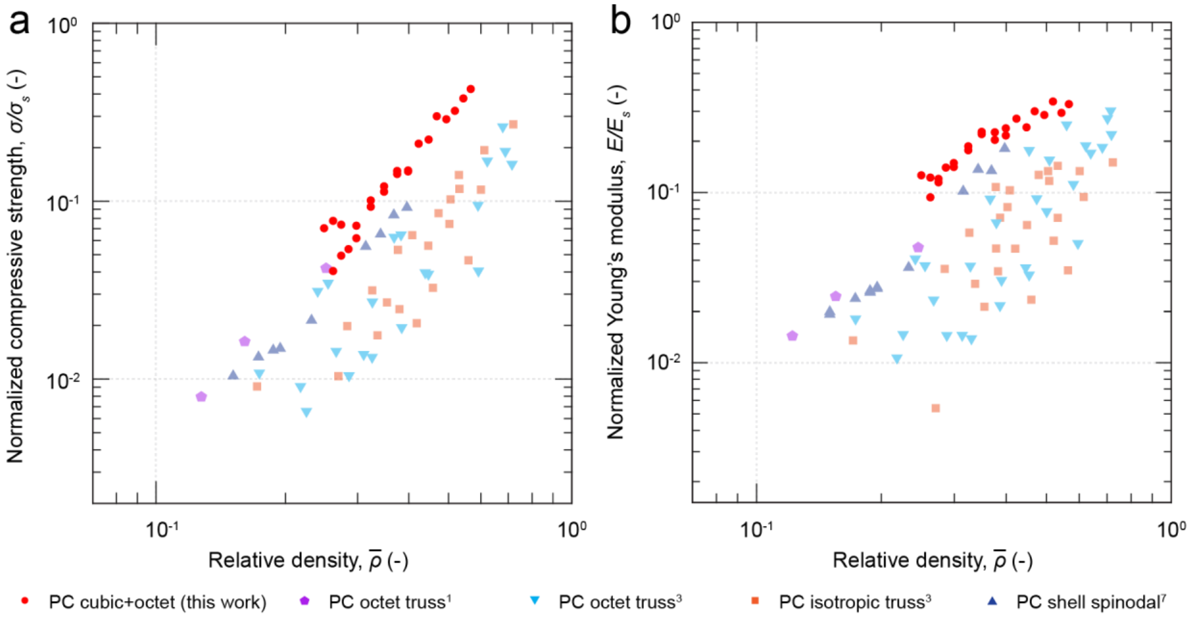

Supplementary Figure 8 | Normalized Ashby charts comparing the cubic+octet plate-architecture to beam and shell topologies in strength (a) and stiffness (b). Source data are provided as a Source Data file.

Supplementary Table 4 | Scaling relations of effective yield strength and stiffness versus relative density of the respective forms $E = BE_s\bar{\rho}^b$ and $\sigma_y = C\sigma_{y,s}\bar{\rho}^c$ given the effective Young's modulus (E) and yield strength (σ_y), the constituent material's Young's modulus (E_s) and yield strength ($\sigma_{y,s}$), the geometric parameters (B and C), the scaling exponents (b and c), and the relative density ($\bar{\rho}$).

Sample Range	BE_s(GPa)	b	$C\sigma_{y,s}$ (GPa)	c
$\bar{\rho} < 37.5\%$)	140.2	2.22	11.4	2.78
$\bar{\rho} \geq 37.5\%$)	38.8	1.05	1.7	0.85

Supplementary Note 3: Computational modeling of cubic+octet plate-lattices

To account for uncertainty in the Poisson's ratio of nanoscale pyrolytic carbon, finite element computations were performed with Poisson's ratios of 0.17[9] and 0.3[10]. No significant change was observed in the results of the normalized Young's Modulus or Zener anisotropy ratio, as shown in Supplementary Figure 9.

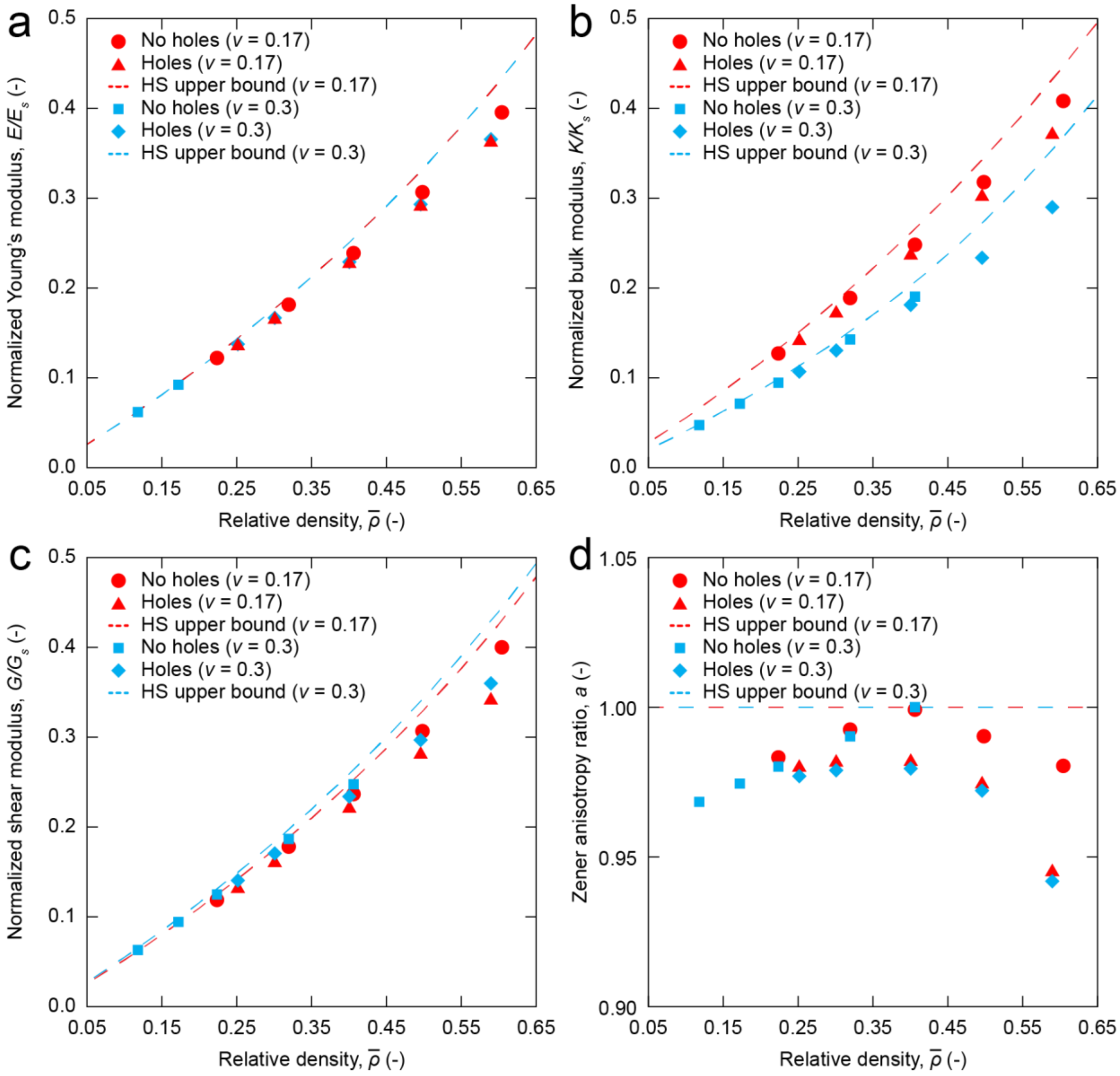

Supplementary Figure 9 | Finite element analysis of cubic+octet plate-lattice performance, for two values of the Poisson's ratio, v = 0.17 and 0.3. Normalized Young's modulus (a), normalized bulk modulus (b), normalized shear modulus (c), and Zener anisotropy ratio (d) versus relative density ($\bar{\rho}$) for models with and without holes. Lines, denoting the Hashin-Shtrikman (HS) upper bound, and points in red and blue represent simulations performed with of 0.17 and 0.3, respectively, and show that independent from v, holes induce no significant knockdown on the Young's modulus or Zener anisotropy ratio, despite noticeable effects on the shear and bulk moduli. Source data are provided as a Source Data file.

Supplementary Figure 10 shows the computed mechanical properties of shell and solid models of ideal plate-lattices do not differ noticeably for most of the investigated relative density range. Simulations with pre-deformed low-$\bar{\rho}$ models and eigenmode analysis to determine the buckling strengths shown in Figure 5 were carried out with shell models. Furthermore, face holes do not significantly alter the buckling strength of ideal shell model plate-lattices below $\bar{\rho} = 50\%$ (Supplementary Figure 10b).

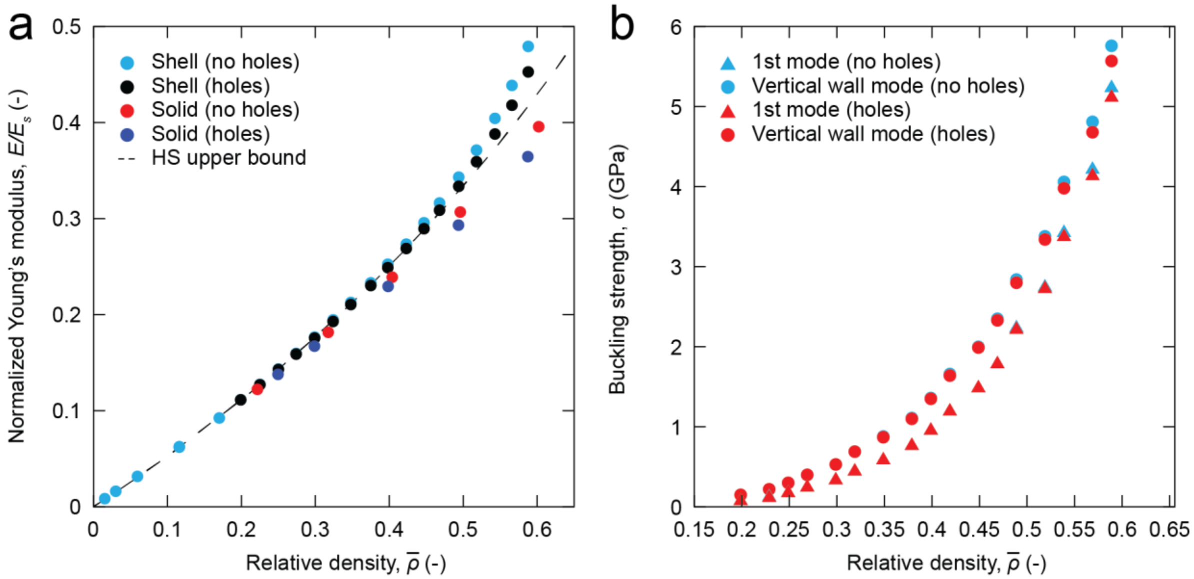

Supplementary Figure 10 | Ideal shell and solid models provide nearly identical results simulating plate-lattices with and without holes up to $\bar{\rho} = 40\%$ (a) and holes do not significantly change the simulated buckling strength of shell model plate-lattices below $\bar{\rho} = 50\%$ (b).

Supplementary Note 4: Nonlinear least square fitting procedure to approximate the constituent material stiffness of pyrolytic carbon cubic+octet plate-nanolattices with face holes

The theoretical upper bound for the Young's modulus of an isotropic cellular material (the Hashin-Shtrikman bound[11]) is given by:

$$\bar{E}_{HSU} = \frac{E_{HSU}}{E_s} = \frac{2\bar{\rho}(5\nu - 7)}{13\bar{\rho} + 12\nu - 2\bar{\rho}\nu - 15\bar{\rho}\nu^2 + 15\nu^2 - 27} \qquad (S1)$$

with the Young's modulus (E_s), Poisson's ratio (ν) of the constituent material, and the relative density ($\bar{\rho}$). The cubic+octet plate-nanolattices of this study included face holes to allow trapped monomer to escape during development. Although small, the holes included a certain knockdown to the stiffness. To account for the contributions of the face holes (see Figure 1), we calculate a correction function (C_C) by least squares fitting the normalized Young's moduli of the finite element plate-lattice data from Figure 2 to the equation:

$$\frac{E_{CO}}{E_s} = C_C\bar{E}_{HSU} \qquad (S2)$$

where E_{CO} is the effective Young's modulus of the cubic+octet plate-lattice with face holes and the correction function is taken as:

$$C_C = C_1\bar{\rho}^3 + C_2\bar{\rho}^2 - (C_1 + C_2)\bar{\rho} + 1 \qquad (S3)$$

with the fit parameters C_1 and C_2. As the Poisson's ratio of the constituent material does not have a noticeable effect on the Young's modulus of the lattice (Supplementary Figure 9), it is not considered in C_C. The correction expression is a cubic polynomial subject to the boundary conditions $C_C = 1$ at $\bar{\rho} = 0$ and $\bar{\rho} = 1$ given that for relative densities of 0% and 100%, e.g. a solid cube, the effective Young's modulus must be 0 and that of the constituent material, respectively. The fitted parameters are therefore found to be $C_1 = 0.92$ and $C_2 = -0.94$. By fitting the following Supplementary Equation 4 with the aforementioned values of C_1 and C_2 and E_s as the only varying fit parameter on the experimentally measured Young's moduli for the plate-nanolattices at $\bar{\rho} \geq 37.5\%$ from Figure 4:

$$E_{CO} = E_s C_{knockdown}\bar{E}_{HSU} \qquad (S4)$$

we extract an effective constituent Young's modulus (E_s) of 62 GPa, in excellent agreement with literature data[12].

Supplementary Note 5: Polymeric cubic+octet plate-microlattices

To investigate the influence of manufacturing-induced plate curvature on the mechanical behavior of low relative density pyrolytic carbon plate-nanolattices, we fabricated and characterized polymeric cubic+octet plate-microlattices with near-ideal, undeformed geometries, complementing our pre-deformed pyrolytic carbon structures (Supplementary Figure 11). Low relative density pyrolytic carbon structures ($\overline{\rho} < 37.5\%$) incurred notable surface deformations during pyrolysis, which were consistent with progressive failure behavior. Here we want to understand whether these imperfections are essential for plate-lattices to manifest progressive failure behavior. The writing parameters of the polymeric plate-microlattices were 25 mW *P* for octet walls, 15 mW *P*, 25 hatched lines and 0.05 µm *hd* for vertical cubic walls, and 23.5 mW *P* and 0.05 µm *hd* for horizontal cubic walls.

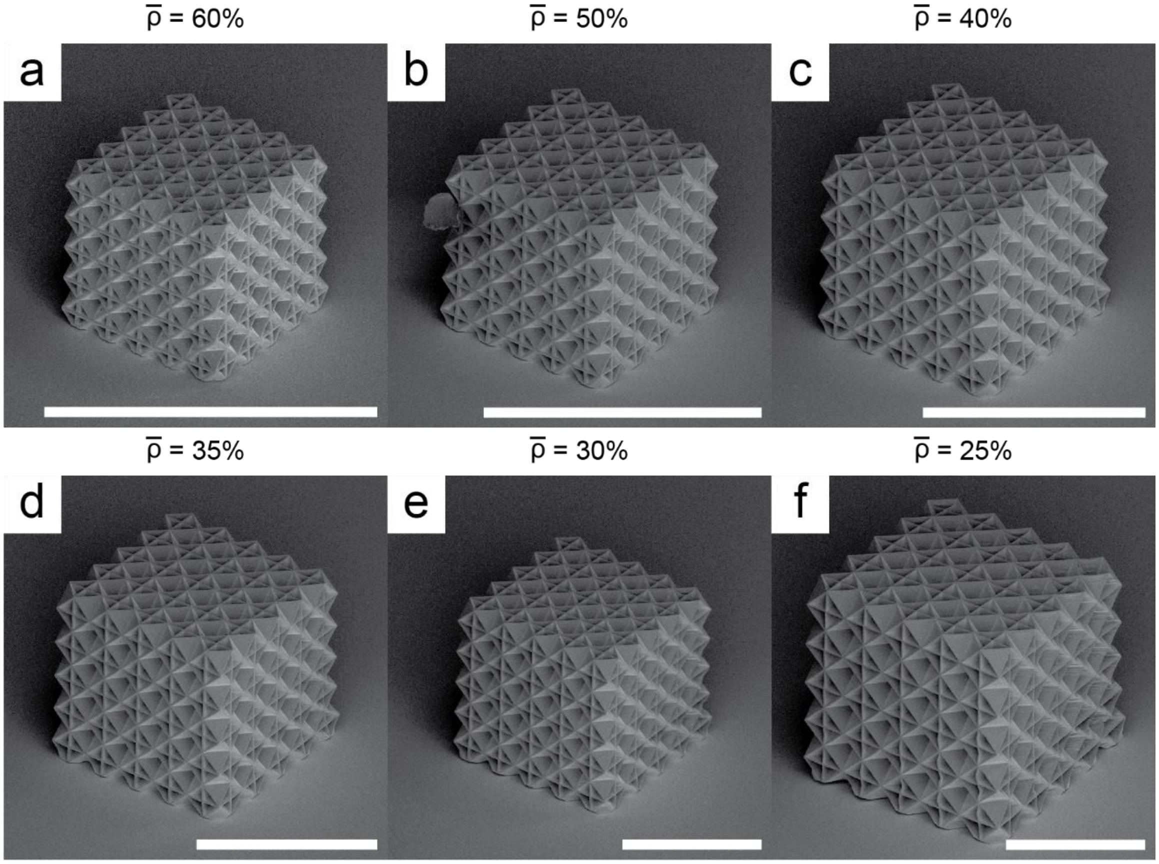

Supplementary Figure 11 | Polymeric plate-microlattices fabricated via TPP-DLW with geometric defect free topologies down to relative densities ($\overline{\rho}$) of 25%. Scale bars are 100 µm.

As in pyrolytic carbon plate-nanolattices, the stress-strain curves of polymer plate-microlattices exhibited a transition from brittle to progressive failure with decreasing relative density (Supplementary Figure 12). Failure in the highest relative density polymer structures ($\bar{\rho} > 40\%$) was catastrophic but became progressive at relative densities of 40% and below, where layer-by layer failure was preceded by buckling of vertical wall members. Except for the lowest relative density polymer lattice ($\bar{\rho} < 25\%$) whose vertical wall members slightly warped during fabrication, buckling was not observed prior to yielding. As for the pyrolytic carbon, polymeric structures were loaded in two cycles where the initial loading and unloading at ~3-4% strain, prior to significant nonlinear behavior, ensured accurate stiffness

measurement. Note that the polymeric lattice of relative density 50% was not tested to failure due to an instrument error. These results show that manufacturing or design-induced pre-curvature of the plate elements is not essential to exhibit progressive failure as long as elastic or plastic buckling can induce sufficient plate curvature preventing catastrophic crack propagation through the entire structure and hence brittle failure[7].

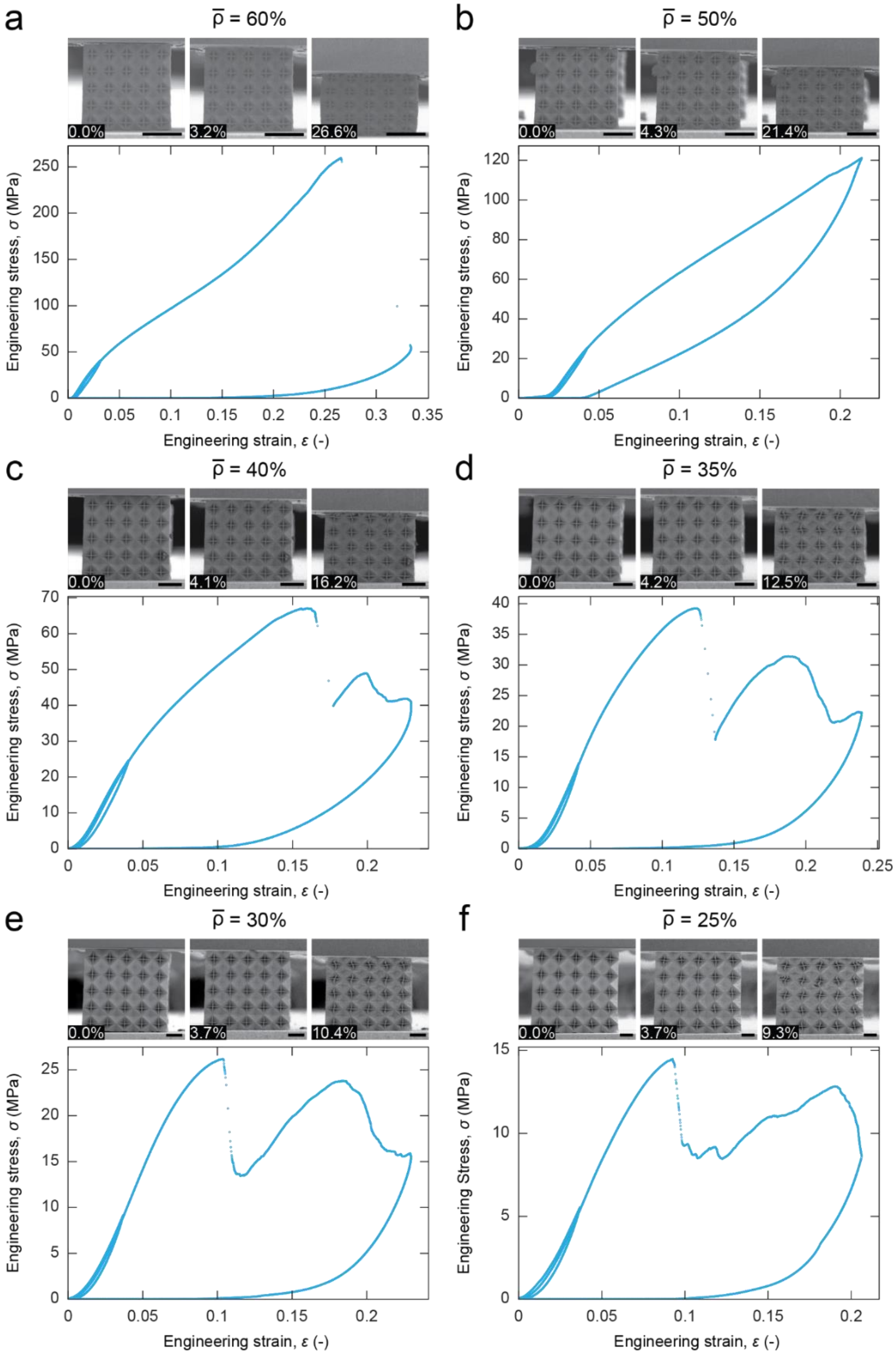

Supplementary Figure 12 | Compression experiments of polymeric cubic+octet plate-microlattices with different relative densities ($\bar{\rho}$). Stress-strain curves of specimens with $\bar{\rho}$ from 60% to 25% (a-f) accompanied by front facing *in situ* SEM images at indicated strains, show transitions from brittle fracture to progressive deformation behavior around 40% relative density. Scale bars are 25 µm. Source data are provided as a Source Data file.

All polymeric lattices lie at the Suquet upper bound for strength (Supplementary Figure 13) except for the lowest relative density sample ($\bar{\rho} = 25\%$), which failed by elastic buckling prior to yielding. Although the constituent polymer material is brittle, measurement challenges related to the viscoelastic nature of the material resulted in drastic knockdowns to the measured stiffness with respect to the Hashin-Shtrikman upper bound[13]. Nonetheless, the stiffness data reflects a clear trend with minimal scatter due to the higher geometric fidelity of the polymer plate-microlattices compared to the pyrolytic carbon ones. Both stiffness and strength did not show a knockdown over the transition from brittle to progressive failure. The shaded regions in Supplementary Figure 13 are enclosed by the upper bounds for stiffness and strength of a constituent material with the minimum and maximum constituent material properties of the polymer, as measured from uniaxial compression of five aspect ratio 3 square pillars with a side length of 20 µm and printed using the vertical cubic wall writing parameters. Viscoelastic polymer properties are sensitive to environmental conditions and measurements showed higher variability than found for pyrolytic carbon. The average polymer Young's modulus and 0.2% offset yield strength were 6.0±2.0 GPa and 67±19 MPa, respectively, whereby higher values may be expected to best represent the constituent properties of the structures.

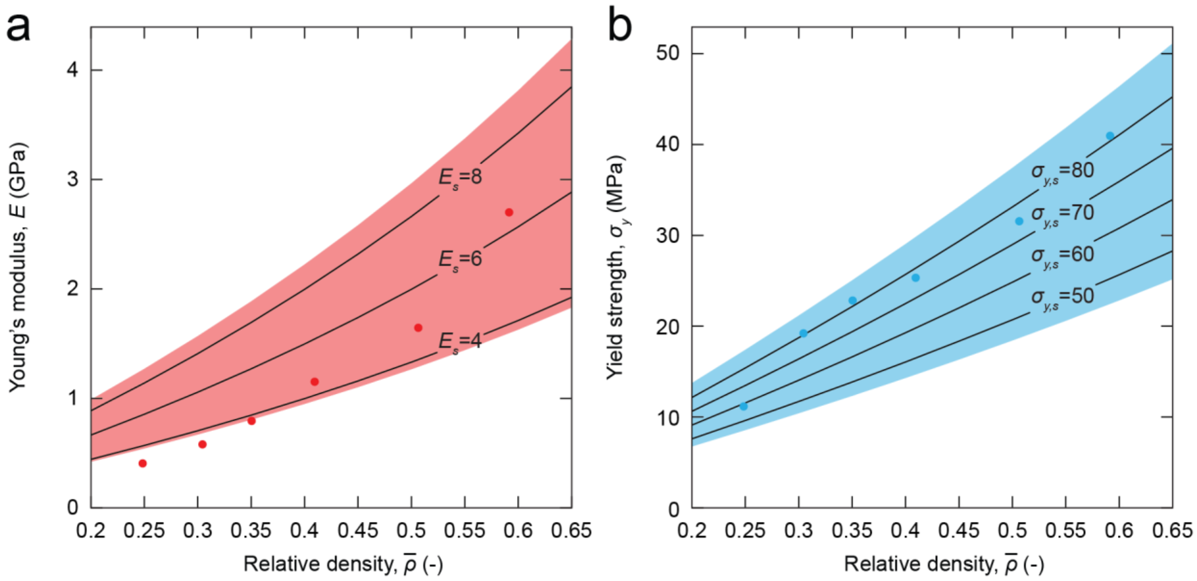

Supplementary Figure 13 | Polymeric cubic+octet plate-microlattices reach the Suquet upper bound for strength of an isotropic cellular material. Stiffness (a) and yield strength (b) versus relative density plots show most data points within the shaded upper bound regions, which are given by the constituent property range of the polymer. Lines (black) indicate upper bound functions for various constituent materials properties. Source data are provided as a Source Data file.

Supplementary References

1. Bauer, J., Schroer, A., Schwaiger, R. & Kraft, O. Approaching theoretical strength in glassy carbon nanolattices. *Nat. Mater.* **15**, 438–443 (2016).
2. Bauer, J. *et al.* Nanolattices: An Emerging Class of Mechanical Metamaterials. *Adv. Mater.* **29**, 1–26 (2017).
3. Zhang, X., Vyatskikh, A., Gao, H., Greer, J. R. & Li, X. Lightweight, flaw-tolerant, and ultrastrong nanoarchitected carbon. *Proc. Natl. Acad. Sci.* 201817309 (2019). doi:10.1073/pnas.1817309116
4. Bauer, J., Guell Izard, A., Zhang, Y., Baldacchini, T. & Valdevit, L. Programmable Mechanical Properties of Two-Photon Polymerized Materials: From Nanowires to Bulk. *Adv. Mater. Technol.* (2019).
5. ASTM International. *Standard Test Method for Compressive Properties of Rigid Plastics*. *Annual Book of ASTM Standards* **i**, 1–8 (2008).
6. Albiez, A. & Schwaiger, R. Size Effect on the Strength and Deformation Behavior of Glassy Carbon Nanopillars. (2018). doi:10.1557/adv.2018.
7. Guell Izard, A., Bauer, J., Crook, C., Turlo, V. & Valdevit, L. Ultrahigh Energy Absorption Multifunctional Spinodal Nanoarchitectures. *Small* **15**, 1–8 (2019).
8. Bauer, J. *et al.* Additive Manufacturing of Ductile, Ultrastrong Polymer-Derived Nanoceramics. *Matter* 1–10 (2019). doi:10.1016/j.matt.2019.09.009
9. Zhao, J. X., Bradt, R. C. & Walker, P. L. J. The Fracture Toughness of Glassy Carbons at Elevated Temperatures. *Carbon N. Y.* **23**, 15–18 (1985).
10. Berger, J. B., Wadley, H. N. G. & McMeeking, R. M. Mechanical metamaterials at the theoretical limit of isotropic elastic stiffness. *Nature* **543**, 533–537 (2017).
11. Hashin, Z. & Shtrikman, S. A variational approach to the theory of the elastic behaviour of multiphase materials. *J. Mech. Phys. Solids* **11**, 127–140 (1963).
12. Manoharan, M. P., Lee, H., Rajagopalan, R., Foley, H. C. & Haque, M. A. Elastic Properties of 4-6 nm-thick Glassy Carbon Thin Films. *Nanoscale Res. Lett.* **5**, 14–19 (2010).
13. Tancogne-Dejean, T., Diamantopoulou, M., Gorji, M. B., Bonatti, C. & Mohr, D. 3D Plate-Lattices: An Emerging Class of Low-Density Metamaterial Exhibiting Optimal Isotropic Stiffness. *Adv. Mater.* **1803334**, 1–6 (2018).
